# A Case of Esophageal Plasmablastic Lymphoma With a Literature Review

**DOI:** 10.7759/cureus.19664

**Published:** 2021-11-17

**Authors:** Mohamad F Ayas, Zayd Ayas, Haidy Elazzamy, Mohammed Barawi

**Affiliations:** 1 Internal Medicine, Ascension St. John Hospital, Detroit, USA; 2 Basic Sciences, University of Texas Austin, College of Natural Sciences, Austin, USA; 3 Pathology and Laboratory Medicine, Ascension St. John Hospital, Detroit, USA; 4 Gastroenterology, Ascension St. John Hospital, Detroit, USA

**Keywords:** esophageal plasmablastic lymphoma, ebv, hiv, diffuse large b-cell lymphoma, extraoral plasmablastic lymphoma

## Abstract

Plasmablastic lymphoma is a rare B-cell lymphoma that is mainly associated with immunocompromised patients, such as those affected with human-immunodeficiency virus (HIV) or those who have received solid organ transplants; however, it has also been documented in immunocompetent patients. Moreover, there is also a strong association with Epstein-Barr virus (EBV). Although mainly found in the oral cavity, a few cases were documented to affect the gastrointestinal tract, and of those, only three cases were found to involve the esophagus, and we present the fourth case in a 48-year-old HIV-positive male.

## Introduction

Plasmablastic lymphoma (PbL) has been recognized by the World Health Organization (WHO) classification as a distinct subtype of diffuse large B-cell lymphoma (DLBCL), which is characterized by a strong expression of plasma cell markers instead of conventional B-cell markers [1,2]. Although strongly associated with HIV-positive patients, it has also been commonly described in patients with solid organ transplants as well as immunocompetent patients [3]. PbL has been shown to have an increased affinity for the oral cavity, followed by the digestive tract [3,4]. Furthermore, tumor involvement of the digestive tract accounts for around 20% of plasmablastic lymphomas [5]. We hereby present an extremely rare case of an esophageal plasmablastic lymphoma in a 48-year-old newly diagnosed HIV male.

## Case presentation

A 48-year-old man with a past medical history of hypertension, alcohol dependence, macrocytic anemia (secondary to a well-established clinical entity of monoclonal gammopathy associated with B-cell lymphoma, with an elevated IgG kappa level of 1380 mg/dL on immunoelectrophoresis), and a smoking history presents to the emergency department with multiple complaints including severe weight loss, poor appetite, fatigue, and nonproductive cough with sinus drainage for the past month. According to the patient, he has lost around 50 pounds in the span of four to five months and has been complaining of early satiety for the past two months. The patient denied any abdominal pain, dysphagia, or odynophagia and denies any history of esophagogastroduodenoscopy (EGD) or colonoscopy in the past. The patient admits to drinking more than one pint of hard liquor a day for the past four years and has been smoking half a pack a day for the past 30+ years. The patient had no family history of cancer. The patient is a homosexual and has multiple sexual partners and does not use protection. During his visit to the emergency department, the patient had two episodes of vomitus with large bright red blood; the patient denies any history of varices or variceal bleeding. 

**Figure 1:**
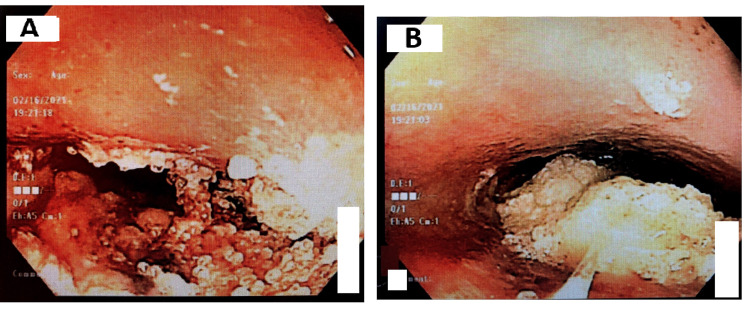
(A and B) EGD showing a friable, ulcerated circumferential mass lesion in the distal esophagus EGD, Esophagogastroduodenoscopy.

Initially, the patient had a WBC count of 10.37 k/mcl (Normal: 4-11) with a hemoglobin of 4.8 g/dL (Normal: 13.5-17.5) and MCV of 117.5 fl (Normal: 80-100) with a platelet count of 72 k/mcl (Normal: 150-450). The patient's creatinine was 2.64 mg/dl (Normal: 0.70-1.5) with an unknown baseline with a sodium of 124 mmol/L (Normal: 135-145), potassium 3.1 mmol/L (Normal: 3.5-5.4), and alkaline phosphatase of 226 IU/L (Normal: 20-130) with AST of 94 IU/L (Normal: 0-45), and a total protein of 9.8 g/dL (Normal: 6.2-8.1). The patient's urine drug analysis was negative and tested negative for coronavirus/SARS-CoV-2 (COVID-19). Vital signs show a heart rate of 120 beats/minute and were afebrile, and the rest of the vital signs were within normal limits. Physical exam showed that the patient was cachectic but not in acute distress. No enlarged lymph nodes were appreciated. Cardiovascular examination showed S1 plus S2 with no audible murmurs, and lungs were clear to auscultation bilaterally. Gastrointestinal examination showed a soft, non-tender abdomen with positive bowel sounds. The patient had 5/5 strength in the upper and lower extremities. Rectal examination showed multiple genital warts, which were coliform in nature, and the guaiac test was negative for any bleeding. Initially, the chest x-ray showed no acute disease; however, an abdominal ultrasound showed fatty changes in the liver but was negative for any gallbladder pathology. Computed tomography (CT) scan of the abdomen/pelvis without contrast was initially done and was negative for any acute pathology. The patient was resuscitated with intravenous (IV) fluids and two units of packed red blood cells and was started on octreotide drip for possible variceal bleed. The patient subsequently underwent EGD for his hematemesis, and a friable, ulcerated circumferential mass lesion was seen in the distal esophagus extending from 28 to 40 cm from the incisors with active oozing that was seen from the mass (Figure 1, Panels A and B). 

**Figure 2:**
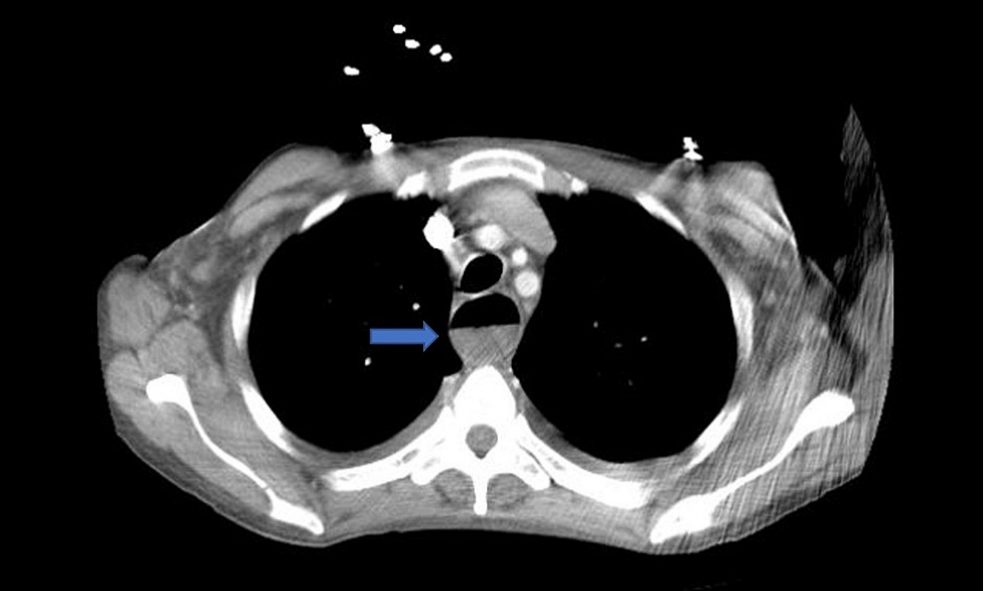
CT chest shows a distended esophagus to about 2.5-3 cm with an air-fluid level (blue arrow)

**Figure 3:**
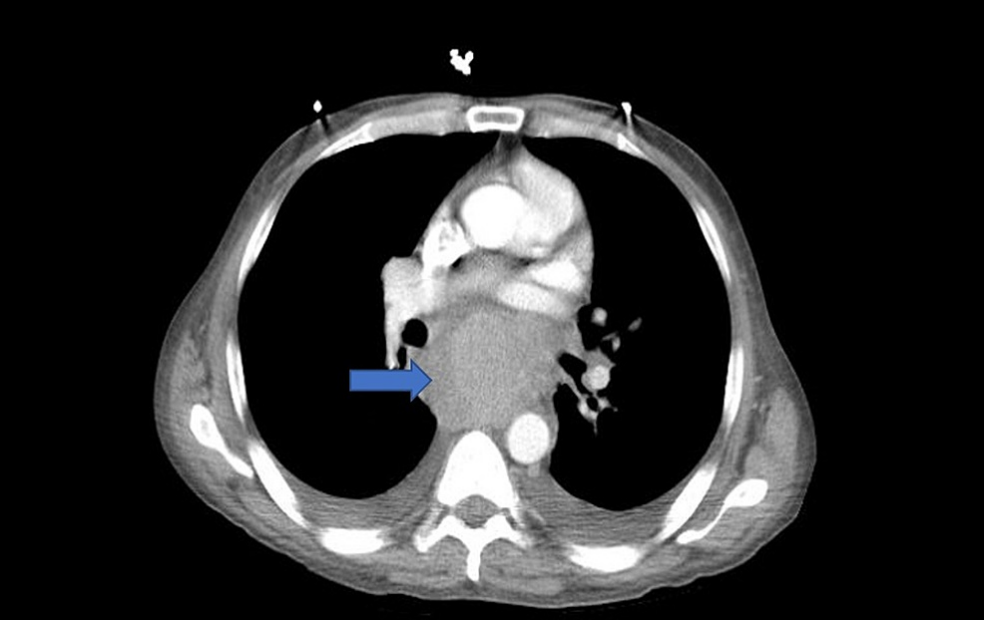
CT chest shows a subcarinal mass-like encirclement of the esophagus with splaying of the carina and mass-effect measuring 7.36 cm x 5.38 cm in its greatest dimensions (blue arrow)

**Figure 4:**
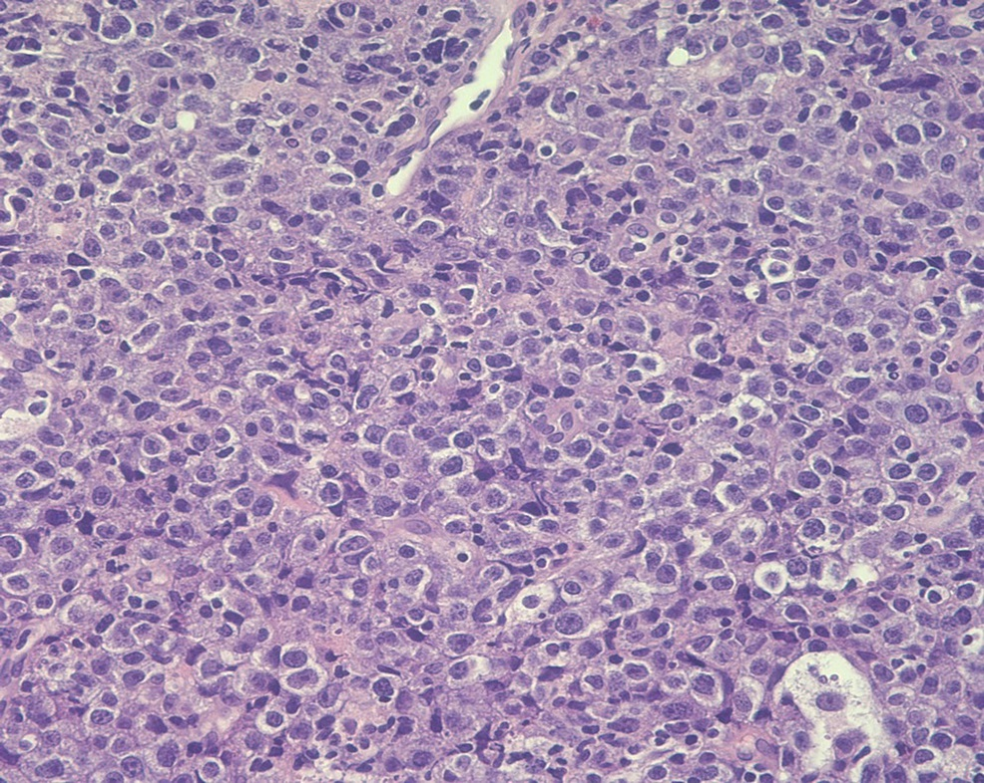
Diffuse proliferation of large, atypical cells associated with brisk mitotic activity and occasional tangible body macrophages (400x esophageal mass) There is immunoblastic morphology with large vesicular nuclei along with single prominent eosinophilic nuclei and abundant finely granular eosinophilic cytoplasm with a relatively mild monotonous appearance and focal starry sky pattern.

**Figure 5:**
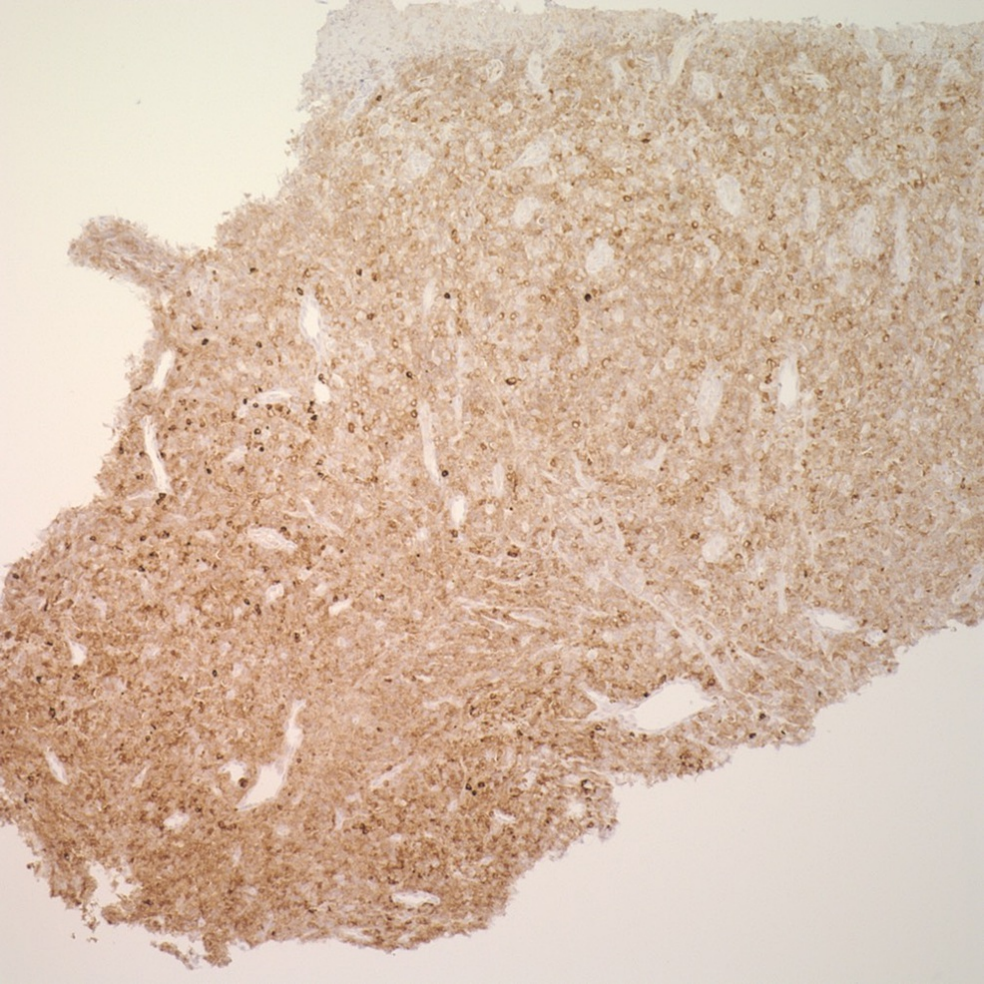
Esophageal mass biopsy positive for CD79a on immunohistochemistry (40x)

**Figure 6:**
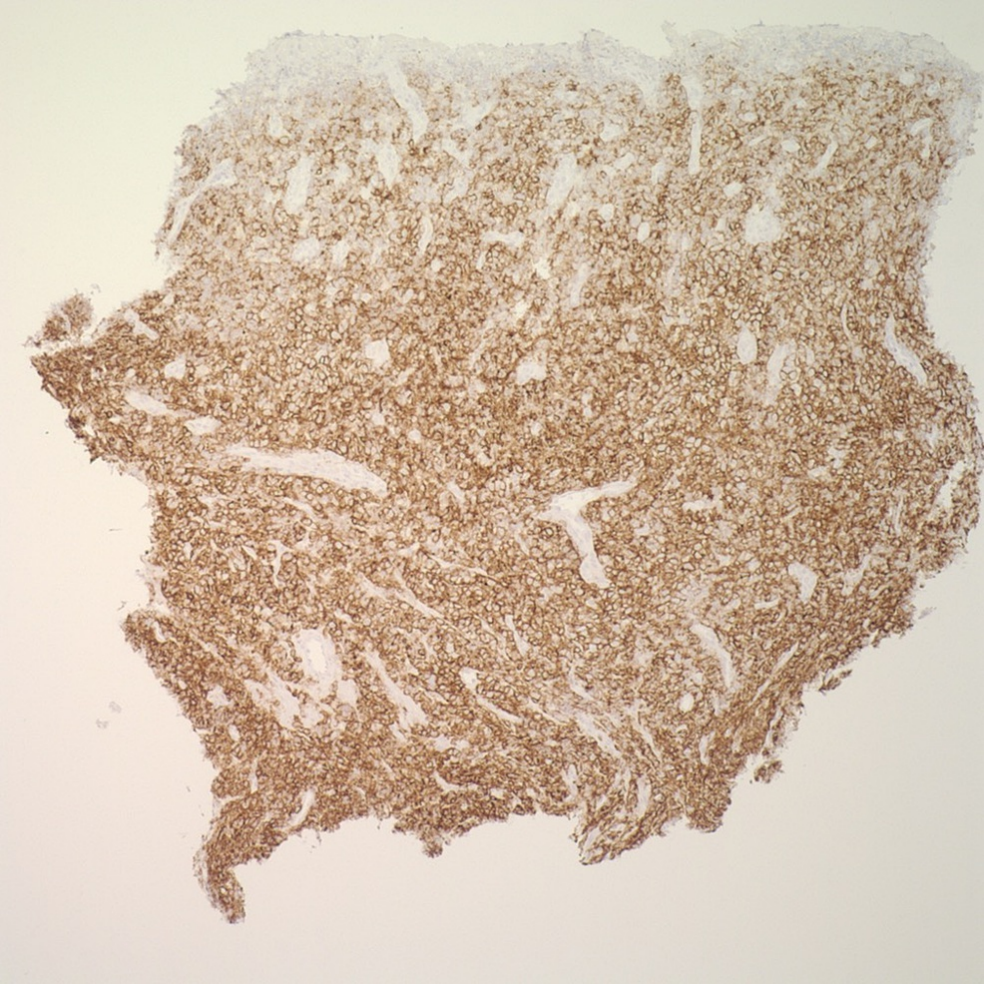
Esophageal mass biopsy positive for CD138 on immunohistochemistry (40x)

**Figure 7:**
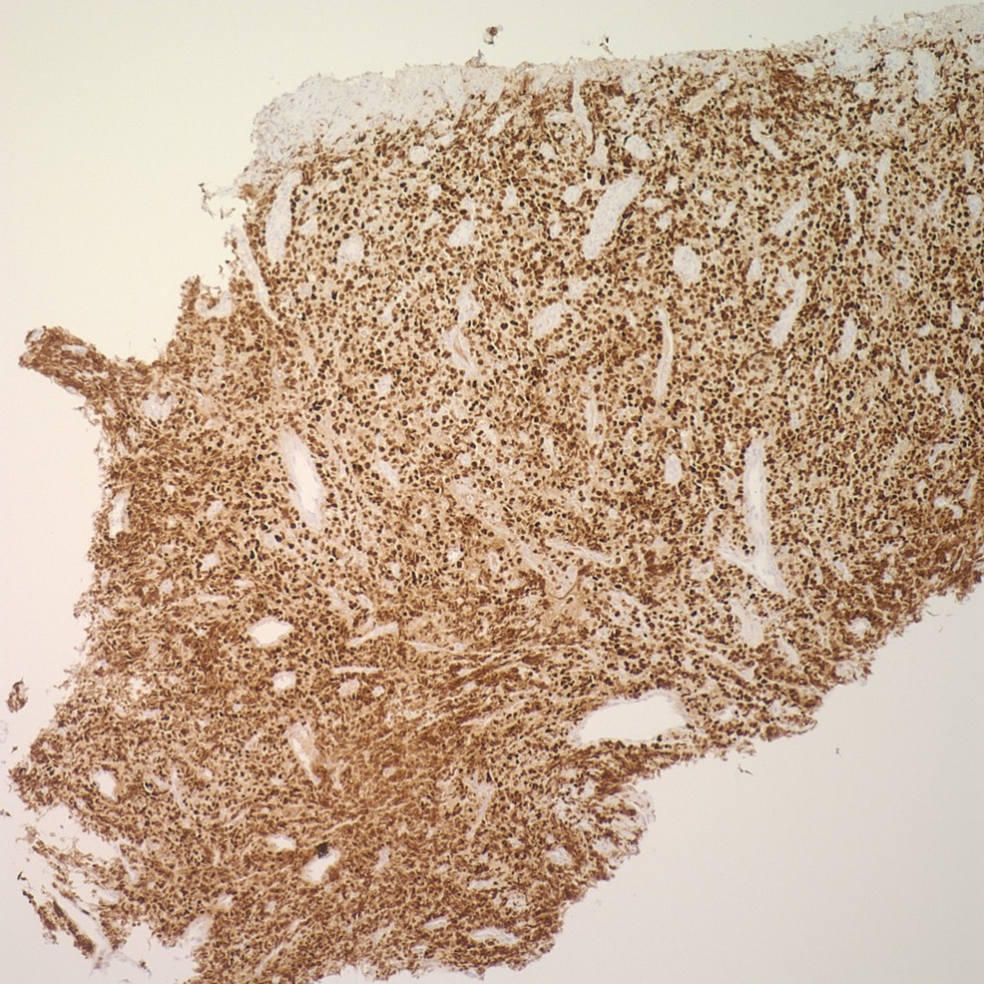
Esophageal mass biopsy positive for MUM-1 on immunohistochemistry (40x)

**Figure 8:**
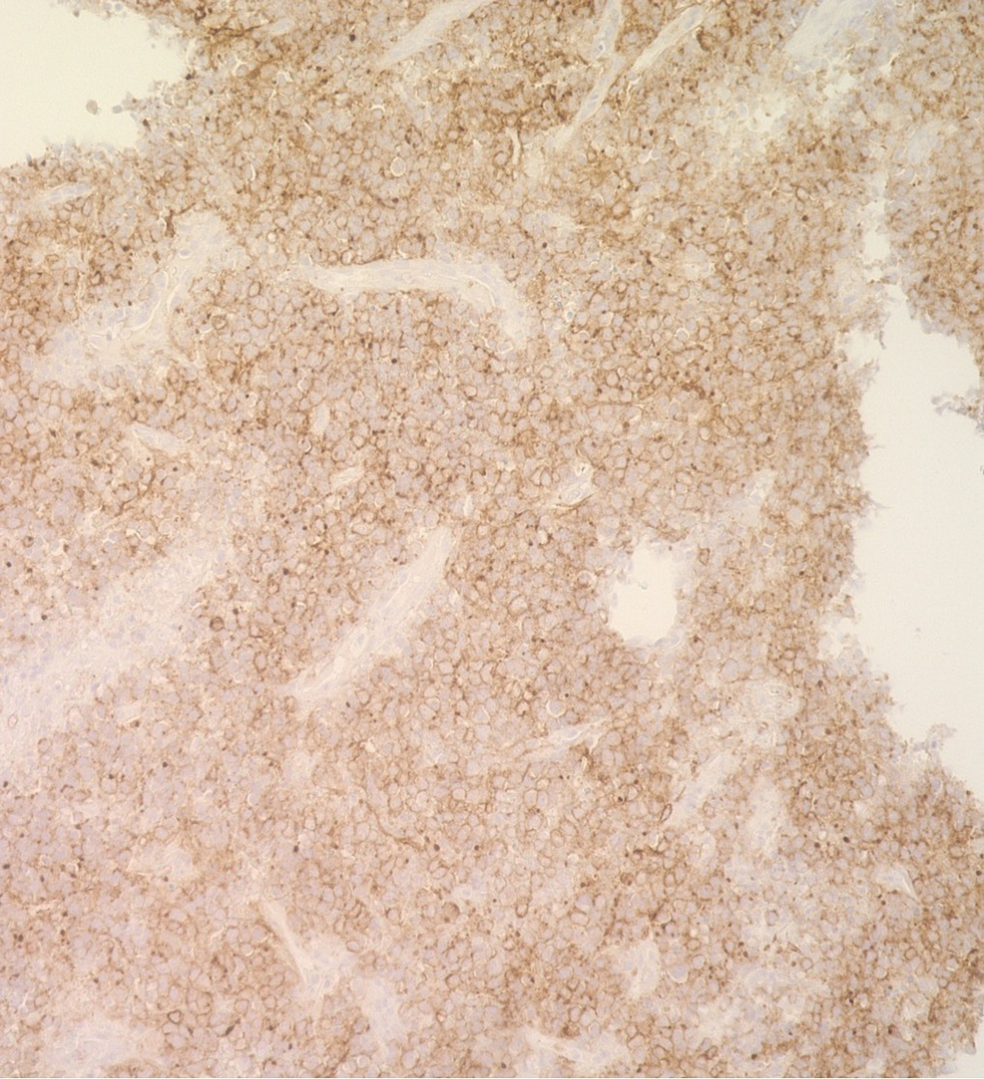
Esophageal mass biopsy positive for CD10 on immunohistochemistry (40x)

**Figure 9:**
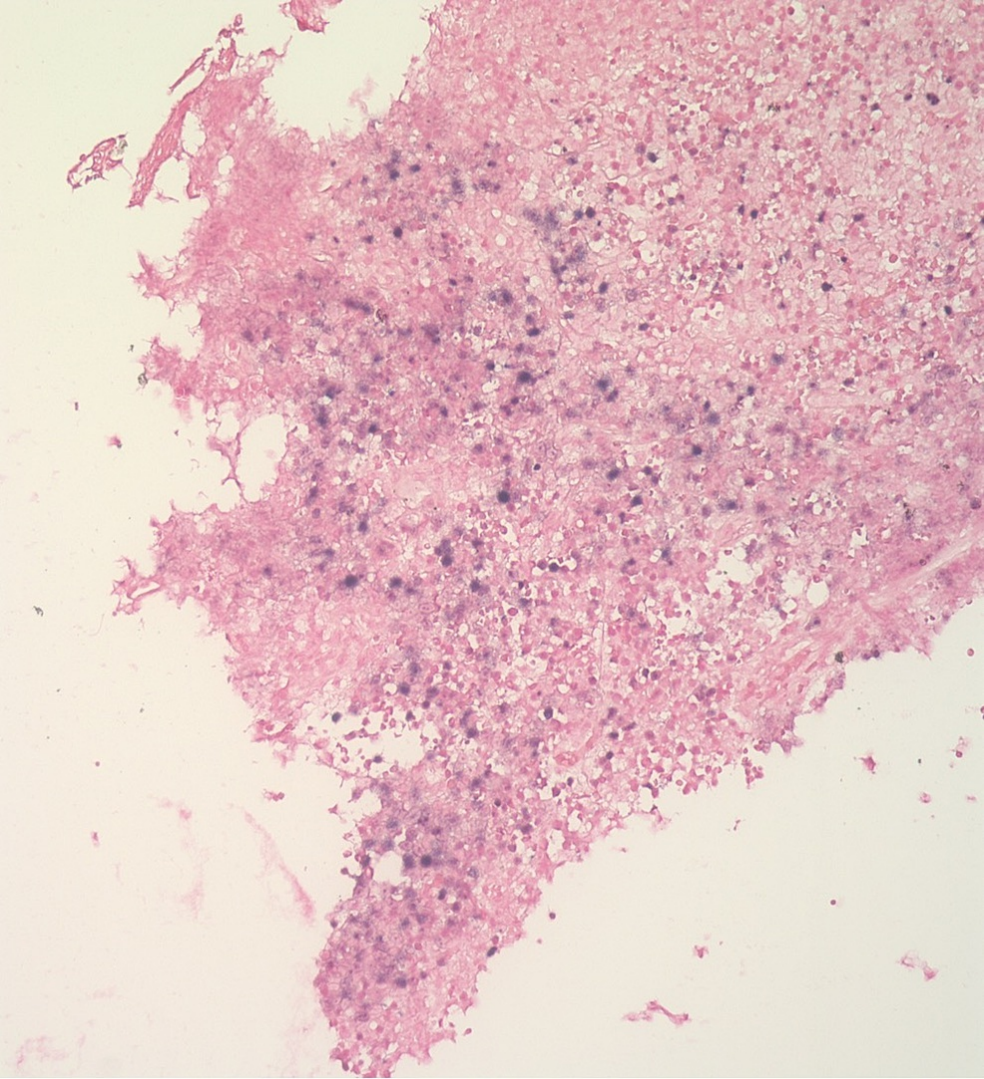
Esophageal mass biopsy positive for EBV in-situ hybridization (40x) EBV, Epstein-Barr virus.

Multiple biopsies were obtained and sent to pathology. Oncology was subsequently consulted. Moreover, the patient’s syphilis screen was nonreactive, he had a normal thyroid-stimulating hormone (TSH) level, hepatitis panel was negative, and tested negative for cytomegalovirus (CMV). Furthermore, the patient was tested for HIV which was positive with a viral load of 219,000 cpy/mL, with appropriate HIV 1 genotyping. The absolute lymphocyte number was 0.93 k/mcl with an absolute CD4 T-helper count of 24 /mm^3^. The patient subsequently underwent CT of the chest abdomen pelvis with IV contrast, which demonstrated a distended esophagus to about 2.5-3 cm with an air-fluid level (Figure 2) and a subcarinal mass-like encirclement of the esophagus with splaying of the carina and mass-effect along the left atrium and inferior pulmonary veins, measuring 7.36 x 5.38 cm in the greatest transverse and anterior/posterior dimensions (Figure 3). Pathologic analysis of esophageal biopsy showed a diffuse perforation of the large, atypical cells associated with brisk mitotic activity consistent with plasmablastic lymphoma (Figure 4). Immunohistochemical (IHC) stains were positive for CD79a, CD138, MUM-1, and CD10 (Figures 5-8, respectively) and were negative for CD20, PAX-5, CD3, CD5, BCL6, BCL2, cyclin D1, CD30, anaplastic lymphoma kinase (ALK), and epithelial membrane antigen (EMA). C-MYC by immunohistochemistry was positive and in-situ hybridization for Epstein-Barr virus (EBV) was positive (Figure 9). The patient also underwent a bone marrow biopsy, which showed hypercellular marrow with 90% cellularity and decreased trilineage hematopoiesis, plasma cells, histiocytes, and lymphocytes. Immunohistochemistry showed the CD-79a highlights with scattered B cells and numerous plasma cells, which were also highlighted by CD-138. Flow cytometry showed CD45-positive lymphocytes and was negative for CD20, CD10, CD 200, CD5, and lambda light chain. The patient was started on chemotherapy with dose-adjusted etoposide, prednisolone, vincristine, cyclophosphamide, doxorubicin (V-EPOCH) and highly active antiretroviral therapy (HAART) with Truvada and Dolutegravir. The patient underwent a total of six cycles of V-EPOCH with dose-adjusted cyclophosphamide due to elevated bilirubin and low CD4 counts. Therapy was complicated by multiple episodes of febrile neutropenia requiring hospitalization and severe peripheral neuropathy requiring multiple dose adjustments; however, the patient tolerated therapy generally well and is now discussing the option of allogeneic bone marrow transplantation.

## Discussion

First described in 1997 by Delecluse et al. [6], PbL was initially identified in the oral cavity and characterized as a rare lymphoma with poor outcome [4]. Most commonly described in the oral cavity, PbL also affects sites such as the gastrointestinal tract, lymph nodes, skin, bones, genitourinary tract, nasal, and paranasal sinuses [1,3,7]. PbL can also affect the gastrointestinal tract starting from the esophagus all the way down to the anorectal region. A PubMed search was performed with the keyword “Esophageal Plasmablastic Lymphoma”, and of the five articles that appeared, only three cases have been reported to involve the esophagus [1,5,8], and we present this case as the fourth. Though the underlying cause is not yet completely known, data suggests that there is a relationship between PbL and immunodeficiency, and it accounts for 2.6% of all HIV-related neoplasms [9]. Moreover, it has been described in HIV-negative patients, and therefore, according to Lopez et al., PbL is classified into three groups based on immune status: HIV-positive patients, post-transplant patients (especially those of solid organ transplantations), and immunocompetent patients [3,10]. Overall, the majority of PbL patients were males and HIV-positive [11], with the mean age of the presentation as 39 years in HIV-positive patients and 58 in HIV-negative patients (11).

According to Mani et al. [1], PbL is divided into three distinct categories: (1) PbL variant localized to the oral mucosa with possible nodal or extranodal sites, with minimal or no plasmacytic differentiation; (2) PbL distinguished by plasmacytic differentiation and extraoral presentation; and (3) PbL associated with HHV-8 and multicentric Castleman disease.

Moreover, in-situ hybridization of EBV infection was found in 60%-100% of all PbL cases [1] and in more than 75% of HIV-positive cases [4,7,9]. Although patients with chronic HBV infection are more likely to develop non-Hodgkin lymphoma, it was not found to be demonstrated in specific to PbL cases [1]. Despite resembling B-cell immunoblasts, PbL most commonly shows a lack of B-cell markers such as CD20 and is associated with a plasma cell immunophenotype and stains positively for plasma cell markers such as CD138 [1]. Moreover, MYC arrangement was also theorized to possibly create a more aggressive disease state as it may have led to the plasmablastic morphology [11]. Treatment of PbL is primary with the use of chemotherapy in combination with HAART therapy which has shown a trend toward better survival in patients with HIV-associated PbL [7,11]. According to Castillo et al., those who were not treated with chemotherapy inevitably died with a median survival of three months [11]. 

Cyclophosphamide, doxorubicin, vincristine, and prednisone (CHOP) have been used in many patients with PbL; however, due to minimal response and decrease survival rates, the National Comprehensive Cancer Network (NCCN) guidelines recommended against CHOP therapy [11] and recommended more intensive regimen such as hyper-CVAD-MA, CODOX-M/IVAC, or EPOCH (infusional) therapy [3,11]. Due to the lack of CD20 expression by PbL cells, the use of anti-CD20 monoclonal antibody therapy is not currently a standard therapy [11]. Unfortunately, over 55% of PbL patients are at stage IV during diagnosis in extraoral as compared to oral sites, indicating more dissemination in extraoral PbL [9]. The median overall survival (OS) rate is 14 months with a five-year OS rate of 31% in HIV-infected patients [11,12] and a median survival rate of nine months in non-HIV PbL cases [11]. As seen in our comparison table of the three cases in the literature describing esophageal plasmablastic lymphoma in combination with our case (Table 1), the mean age of diagnosis in those with esophageal PbL was 45 years of age, and 100% of those patients were males. Three of four (75%) were HIV-positive, and of those tested, two of three (67%) were positive for EBV. Four of four (100%) all tested positive for CD138 plasma cell marker, and three of four (75%) of those tested were negative for CD20 B-cell marker.

**Table 1:** Comparison table of esophageal PbL cases HIV, Human-immunodeficiency virus; EBV, Epstein-Barr virus; M, Male; N/A, no answer.

#	Study	Year	Age	Sex	HIV status	EBV status	CD-138 plasma cell marker	CD-20 B-cell marker	References
1	Mani et al.	2008	40	M	+	+	+	-	[1]
2	Mihara et al.	2016	43	M	-	-	+	-	[5]
3	Abdulla et al.	2019	49	M	+	N/A	+	N/A	[8]
4	Ayas et al.	2021	48	M	+	+	+	-	N/A

## Conclusions

Esophageal plasmablastic lymphoma is an extremely rare and aggressive subtype of DLBCL and is most commonly found in HIV-positive patients. Disease awareness and early detection are warranted, especially for those with risk factors such as smoking, alcohol use, and HIV with concurrent B symptoms as 55% of extraoral PbL patients was found to be at stage IV during diagnosis.
